# Estimating Resting HRV during fMRI: A Comparison between Laboratory and Scanner Environment

**DOI:** 10.3390/s21227663

**Published:** 2021-11-18

**Authors:** Andy Schumann, Stefanie Suttkus, Karl-Jürgen Bär

**Affiliations:** Lab for Autonomic Neuroscience, Imaging and Cognition (LANIC), Department of Psychosomatic Medicine and Psychotherapy, Jena University Hospital, 07740 Jena, Germany; stefanie.suttkus@med.uni-jena.de (S.S.); karl-juergen.baer@med.uni-jena.de (K.-J.B.)

**Keywords:** heart rate variability, autonomic nervous system, reproducibility, photoplethysmogram, electrocardiogram

## Abstract

Heart rate variability (HRV) is regularly assessed in neuroimaging studies as an indicator of autonomic, emotional or cognitive processes. In this study, we investigated the influence of a loud and cramped environment during magnetic resonance imaging (MRI) on resting HRV measures. We compared recordings during functional MRI sessions with recordings in our autonomic laboratory (LAB) in 101 healthy subjects. In the LAB, we recorded an electrocardiogram (ECG) and a photoplethysmogram (PPG) over 15 min. During resting state functional MRI, we acquired a PPG for 15 min. We assessed anxiety levels before the scanning in each subject. In 27 participants, we performed follow-up sessions to investigate a possible effect of habituation. We found a high intra-class correlation ranging between 0.775 and 0.996, indicating high consistency across conditions. We observed no systematic influence of the MRI environment on any HRV index when PPG signals were analyzed. However, SDNN and RMSSD were significantly higher when extracted from the PPG compared to the ECG. Although we found a significant correlation of anxiety and the decrease in HRV from LAB to MRI, a familiarization session did not change the HRV outcome. Our results suggest that psychological factors are less influential on the HRV outcome during MRI than the methodological choice of the cardiac signal to analyze.

## 1. Introduction

Heart rate variability (HRV) is an established marker of all-cause morbidity and mortality [1,2]. Furthermore, HRV has been shown to respond to emotional and cognitive stimulation [3,4,5]. For this reason, the central correlates of cardiac activity and its relation to emotions and cognition seem to be an important focus of current research in functional neuroimaging [6,7]. Crucial findings have shed light on the association of HRV with cortical structures [8,9,10] or function [11,12,13]. In particular, functional magnetic resonance imaging (fMRI) with concurrent recordings of physiological signals has facilitated the identification of a network of brain regions that are involved in the central control of the heart [7,14,15]. These findings have corroborated and extended our understanding of the central autonomic network that was described decades ago mainly based on animal and lesion studies [16].

Resting fMRI has become increasingly popular, as it reveals functional interactions between brain regions under resting conditions without specific stimulation. This unconstrained environment is supposed to allow free mind wandering in participants. However, imaging their brain by fMRI is a rather stressful event for most participants. The scanner is noisy and cramped, and therefore some participants need to terminate the scanning session ahead of time.

To the best of our knowledge, the influence of the fMRI environment on resting HRV has not been systematically investigated. It seems that most researchers assumed HRV measurement in the scanner to be equivalent to laboratory assessments. Additionally, the concordance of HRV estimated in repeated fMRI sessions (stability) remains unclear.

In laboratory settings under carefully controlled resting state conditions, the stability of HRV has been analyzed in various test–retest studies [17,18]. Marks and Lightfoot (1999) investigated the reproducibility of HRV obtained from two trials within a one-week period [19]. The authors reported that all time-domain variables were highly reproducible, with intra-class correlation coefficients (ICCs) of ≥0.84, while frequency-domain variables showed less reproducibility (ICC range 0.67–0.96). Cipryan and Litschmannova (2013) found that the stability of spectral HRV indices decreases when repeating the retest after some days compared to immediate retest without interruption. ICCs estimated in this study ranged between 0.48 and 0.95. Silva and colleagues (2016) investigated the effect of different body positions on the test–retest stability of HRV. The supine position was the most reproducible posture that led to a high stability of short-term HRV with an ICC of >0.93.

During fMRI, it is often more feasible to record photoplethysmograms (PPG) of the finger to obtain HRV indices, as strong electromagnetic fields cause various artifacts on the electrocardiogram recordings (ECG) [20]. Most research groups that acquire cardiac signals in brain imaging studies use the finger pulse instead of ECGs, as revealed by a mega-analysis of neuroimaging data from an international community [8]. Several studies suggested that the pulse rate can be used as an accurate surrogate for the heart rate [21,22,23]. A meta-analysis by Schäfer and Vagedes (2013) concluded on the ‘sufficient accuracy’ of HRV derived from the finger pulse, especially during the resting condition [24]. However, recent studies suggested that pulse rate variability is not concordant with HRV extracted from ECG recordings [25,26]. Therefore, estimating HRV based on PPG signals instead of ECGs might contribute to a difference in resting HRV between laboratory and MRI measurements.

Additionally, it is well known that the heart rate is sensitive to acute mental stress (e.g., see review [27]). It seems obvious that the noisy and cramped space within the scanner might have an impact on the HRV of participants. Additionally, the fear of suffocation or harmful effects of scanning, claustrophobia or concerns about pathological findings might raise anxiety levels and influence the physiological states of subjects [28].

Chapman and colleagues (2010) investigated differences in the mean heart rate of eleven participants during a one-hour scan and compared the results to recordings in their laboratory [29]. To assess the influence of anxiety, the scan was repeated after one week. The results of this study suggested that the heart rate is elevated during MRI sessions only in the first analyzed time interval (0–8 min). After that, heart rates were comparable between MRI and laboratory sessions. The strongest effect was detected between scan and re-scan over the entire one-hour recording. More recently, Pfurtscheller and colleagues (2018) investigated resting HRV during functional MRI repeated on the same day in 23 healthy individuals [30]. Their results indicated that HRV increased from the first to the second scan, while anxiety significantly decreased. However, the authors did not compare these results to measurements in the laboratory.

How psychological stress during fMRI sessions influences resting HRV indices in comparison to recordings in the laboratory is still unclear. In this study, we aimed at comparing resting HRV from ECGs recorded in the laboratory with PPG recordings during an fMRI session that were less than 2 weeks apart. In the laboratory, we acquired ECG and PPG signals simultaneously to compare HRV estimates from both signals. In order to assess the impact of general nervousness, participants were asked to rate their anxiety level. In a subgroup, we repeated MRI and LAB sessions after approximately one week to estimate the effect of habituation on the environment.

## 2. Methods

### 2.1. Study Design and Participants

We assessed physiological data at rest in 101 healthy volunteers (55% females). Healthy subjects had no present or past history of psychiatric, neurological or other clinically significant disorders. For that reason, we took a careful medical history and a full clinical examination of all subjects. All participants gave their informed written consent in accordance with the protocol approved by the Ethics Committee of the University Hospital Jena. From the initial sample, 22 datasets were excluded due to a poor signal quality of the photoplethysmogram (see Signal Preprocessing).

In sample A, 74 healthy volunteers were investigated in our laboratory with recordings of a finger photoplethysmogram and an electrocardiogram (ECG) at rest. Eleven datasets were excluded due to a poor PPG quality. From this sample, 55 subjects had an MRI scan with a PPG recording of sufficient quality. In an independent sample, laboratory recordings as well as fMRI recordings were repeated within one week. All four sessions were conducted within two weeks without a fixed order. After the exclusion of 3 subjects due to PPG quality in the MRI sessions, 24 participants remained in sample B. In this sample, no PPG signals were acquired in the laboratory sessions.

Before each MRI session, participants rated their level of agitation via a self-assessment manikin (SAM, Bradley, 1994) in domains quality (−5 to 5 being negative to positive) and intensity (0 to 10 being not to very intense). As SAM results are rather coarse, they were used only for describing the sample (see Table 1). Additionally, the State–Trait Anxiety Inventory (STAI) was evaluated before each MRI session [31].

### 2.2. Laboratory Measurement Protocol

The examination room was quiet and fully shaded with a low-intensity ambient light source. Additionally, participants wore headphones to be isolated from potential noise from the surroundings. On a monitor fixed over the couch, a dark gray ellipse was displayed on a light gray background as a fixation anchor. Room temperature was controlled at 22 °C. Resting recordings were conducted in the supine position for 15 min. The first five minutes was not analyzed, in order to exclude the adjustment period to the environment.

### 2.3. MRI Measurement Protocol

MRI sessions were conducted in a dark, temperature-controlled room, with participants lying in a 3T scanner PRISMA (Siemens, Erlangen, Germany). First, an anatomical scan was performed lasting 5 min, followed by a 15 min resting state functional scan with concurrent physiological recordings. During this time, participants were instructed to look at a gray ellipse displayed on a screen (the same as presented in the laboratory sessions).

### 2.4. Data Acquisition and Preprocessing

In the laboratory, we used the MP150 system (BIOPAC Systems Inc., Goleta, CA, USA) to record multiple physiological signals simultaneously at a 1000 Hz sampling rate [32]. A one-channel ECG was acquired via three electrodes attached to the chest and amplified between 0.05 and 35 Hz. The photoplethysmogram was recorded via an optical sensor attached to the distal phalanx of the right index finger. The sensor (TSD200, BIOPAC Systems Inc., Goleta, CA, USA) emits infrared light with wavelengths between 800 and 920 nm and measures the amount of reflected light by a photodiode. The voltage output of the photoresistor was amplified between 0.05 and 10 Hz.

During fMRI, we used another MP150 system equipped with an MR-compatible PPG module to acquire PPG signals at a 500 Hz sampling rate and the same filter settings (TSD200-MRI, BIOPAC Systems Inc., Goleta, CA, USA).

### 2.5. Signal Preprocessing

For processing and analysis of physiological signals, we used the free PhysioNet Cardiovascular Signal Toolbox [33] implemented in MATLAB (R2019a, The Mathworks, Natick, MA, USA). Heart beats were extracted from the ECG automatically using the Pan-Tompkins algorithm (jqrs.m). Pulse wave onsets were detected by analyzing the slope sum function (qppg.m). A PPG signal quality index was estimated based on the dynamic beat template correlation (PPG_SQI_buf.m [34]). All signals with quality estimates below 80% were excluded from the analysis.

### 2.6. Estimation of Heart Rate Variability (HRV)

Artifacts and ectopic beats in the beat-to-beat time series were detected and removed using an adaptive filtering technique [35]. The mean heart rate (*HR*) and its variability indices *SDNN* and *RMSSD* were estimated according to the established standard procedures on the $BBI_{i}$ time course with the mean $\bar{BBI}$ [36].

$$\begin{array}{l} HR=\frac{1}{N}\sum_{i}\frac{60}{1000\cdot BBI_{i}}\\ SDNN=\sqrt{\frac{1}{N}\sum_{i}(BBI_{i}-\bar{BBI})²}\\ RMSSD=\sqrt{\frac{1}{N}\sum_{i}(BBI_{i}-BBI_{i+1})²} \end{array}$$

### 2.7. Comparison of HRV Estimates

**Figure 1:**
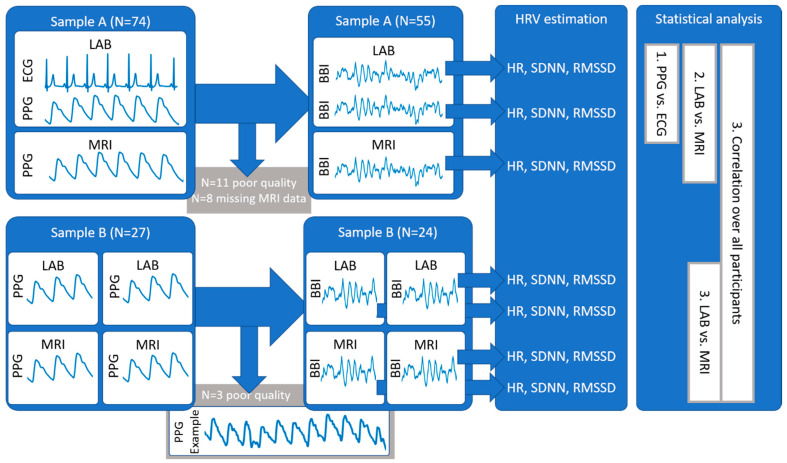
Schematic illustration of the workflow in this study.

In general, we analyzed photoplethysmograms (PPG) and electrocardiograms (ECG) recorded in our laboratory (LAB) and during fMRI sessions (MRI), as depicted in the schematic illustration in Figure 1. Estimated HRV indices were compared using intra-class correlation (two-way mixed effects, consistency, single-rater ICC given with 95% confidence interval). The coefficient of variation (CV) was calculated as the sample standard deviation divided by the sample mean value.

#### 2.7.1. Comparison of HRV Derived from ECG and PPG

First, we investigated the difference between PPG and ECG signals as a methodological influence on HRV results. In the LAB, we recorded both physiological signals simultaneously. HRV derived from both signals was compared in terms of a non-parametric Wilcoxon signed-rank test (N = 55, see Section 3.1). The concordance of HRV parameters between PPG (Y) and ECG signals ($\hat{Y}$) was estimated by means of Pearson correlations and mean absolute errors (*MAE*).

$$MAE=\sum_{i}\left|\hat{Y}-Y\right|$$

#### 2.7.2. Comparison of HRV Recorded in the Autonomic Laboratory and during fMRI

On another day, these 55 participants had an MRI scan with a concurrent finger pulse recording. HRV estimated during MRI (MRI_PPG) was compared to LAB recordings of ECGs and PPGs. A systematic effect of the environment was assessed using a Wilcoxon signed-rank test (N = 55, Section 3.2).

#### 2.7.3. The Effect of Habituation and Anxiety Ratings on HRV Estimated during fMRI

In an independent sample of healthy volunteers (N = 24), we compared two repetitive sessions in the LAB and MRI (T0 and T1). A habituation effect was assessed in both conditions (LAB_ECG_T0 vs. LAB_ECG_T1, and MRI_PPG_T0 vs. MRI_PPG_T1) as well as a remaining difference due to condition after familiarization (LAB_ECG_T1 vs. MRI_PPG_T1) using Wilcoxon signed-rank tests.

A general effect of anxiety prior to the MRI session on the HRV difference between ECG_LAB and PPG_MRI was investigated over all participants using Pearson correlation with STAI state anxiety ratings (Section 3.3).

Results are reported either as a median value and interquartile range (25–75%) or mean ± standard deviation. Statistical significance was assumed at *p* < 0.05. Scatterplots were used for visualization. For statistical analysis, we used IBM SPSS Statistics 27.0 (IBM Corp., Armonk, NY, USA).

## 3. Results

### 3.1. Comparison of HRV Derived from ECG and PPG

In recordings obtained in the autonomic LAB, we first analyzed potential differences in HRV results between PPG and ECG recordings (see Table 2). Mean heart rate (HR) showed the highest level of concordance with a Pearson correlation of r = 0.998. Heart rate variability measures were less consistent when extracted from PPG recordings but still showed high correlations of 0.886 for SDNN and 0.877 for RMSSD. The mean absolute error (MAE) between PPG- and ECG-derived indices ranged from 0.5/min for HR, 5.5 ms for SDNN and 9.1 ms for RMSSD. A Wilcoxon signed-rank test revealed that RMSSD (Z = 5.81, *p* < 0.001) was overestimated when obtained by means of PPG instead of ECG recordings, with an average difference of 12.3%.

**Figure 2:**
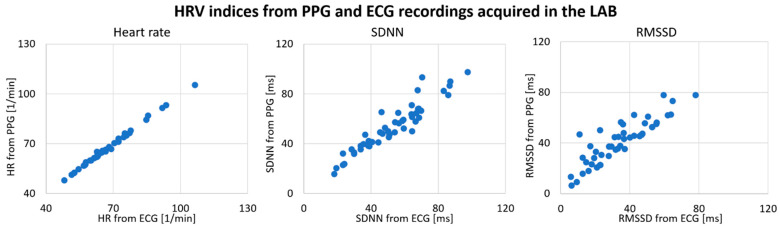
Comparison of HRV from photoplethysmogram and electrocardiogram recorded simultaneously in the laboratory (N = 55). HR: mean heart rate; SDNN: standard deviation of heart beat intervals; RMSSD: root mean square of successive heart beat intervals; ECG: HRV derived from an electrocardiogram recorded in the laboratory; PPG: HRV derived from a photoplethysmogram recorded in the laboratory.

In Figure 2, HRV measures based on PPG and ECG recordings are depicted in scatterplots. The calculated HR showed an almost exact agreement between PPG and ECG, with a Pearson correlation coefficient above 0.99 (Figure 2, left). In particular, RMSSD values deviated in one direction from the equivalence line, showing a tendency to a higher RMSSD when extracted from PPG recordings (Figure 2, right).

### 3.2. Comparison of HRV Recorded in the Autonomic Laboratory and during fMRI

Deriving HRV indices from PPG recordings during MRI sessions seems to be highly concordant with recordings in the LAB (Table 3). Again, HR showed the highest concordance, with an ICC = 0.882, and the lowest CV of all HRV indices. SDNN and RMSSD estimated during MRI showed high correlations with the LAB recordings, with ICCs of 0.8. The Wilcoxon signed-rank test revealed no systematic influence of the MRI environment on any HRV index, independent of the underlying physiological signal.

**Figure 3:**
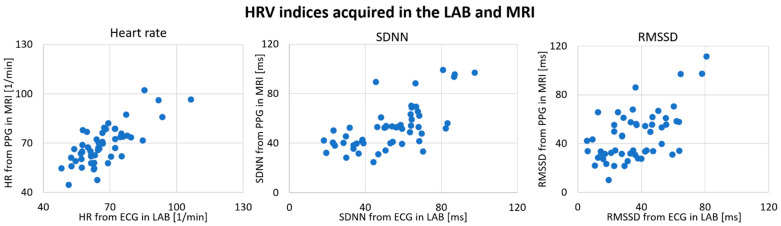
Comparison of HRV from photoplethysmogram recordings during MRI sessions compared with electrocardiogram recordings from the laboratory (N = 55). HR: mean heart rate; SDNN: standard deviation of heart beat intervals; RMSSD: root mean square of successive heart beat intervals; ECG: HRV derived from an electrocardiogram recorded in the laboratory; PPG: HRV derived from a photoplethysmogram recorded during MRI.

In Figure 3, HRV indices from PPGs recorded in the LAB and during MRI are plotted against HRV from ECG LAB recordings.

### 3.3. The Effect of Habituation and Anxiety Ratings on HRV Estimated during fMRI

In an independent sample, we investigated the effect of habituation on resting HRV that was assessed in the laboratory and during MRI. An overview of HRV indices in each session is presented in Table 4. There was no significant difference between the first and second sessions in any of the estimated HRV indices, neither during MRI nor in the LAB. After habituation, there was still a significant difference in HR at T1 between MRI and LAB (Z = 2.591, *p* < 0.05).

**Figure 4:**
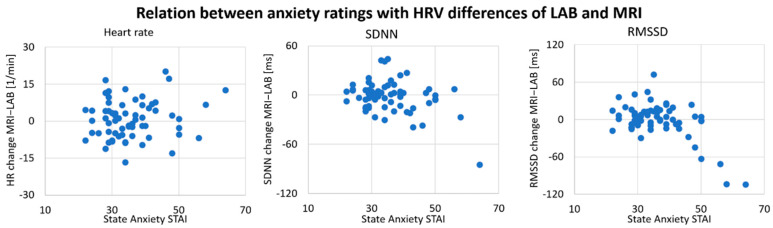
Relationship between anxiety ratings (STAI state anxiety) and HRV differences between LAB (based on ECG) and MRI (based on PPG) over all participants (N = 79). HR: mean heart rate; SDNN: standard deviation of heart beat intervals; RMSSD: root mean square of successive heart beat intervals; MRI: HRV derived from a photoplethysmogram recorded during MRI; LAB: HRV derived from an electrocardiogram recorded in the laboratory.

Differences in HRV between LAB and MRI were correlated with STAI state anxiety levels (Figure 4, SDNN: r = −0.360, *p* < 0.01; RMSSD: r = −0.461, *p* < 0.01), indicating that higher pre-MRI anxiety was associated with stronger decreases in HRV from the LAB to MRI sessions. Differences in HR were not correlated with anxiety ratings (r = 0.142).

## 4. Discussion

This study investigated the stability of time-domain HRV estimates when recordings take place in an MR scanner during brain imaging instead of a standardized assessment in a laboratory. We analyzed influences of the underlying cardiac signal (PPG or ECG) and individual levels of pre-MRI anxiety.

Comparing the environment of MRI with LAB sessions, the loudness and tightness during brain imaging are obvious. In contrast, we found no systematic influence of the environment (LAB vs. MRI) on any HRV index, independent of the underlying physiological signal (PPG or ECG). High ICC values, all above 0.8, suggested that HRV measures were stable across conditions and signals. There does not seem to be a systematic effect of MRI condition on HRV, which one might expect from the noisy and cramped environment during scanning. The scatterplots suggest that there is no tendency to a higher HR and lower HRV in the MR scanner compared to LAB sessions. It seems that subjects reacted differently to the environmental change.

To put our results regarding HRV stability in perspective, test–retest studies that have been performed in the laboratory have only reported similar results (see review by Sandercock et al., 2005 [17]). Using a delay of several days to one week, ICC values between 0.67 and 0.96 [19], or 0.58 and 0.96 [37], have been obtained. Lord and colleagues performed 10 min resting state recordings in two laboratory sessions that were one week apart. In 21 healthy controls, a CV of 45% indicated high variability between HRV estimates [38]. Even when the exact same recordings are analyzed by different researchers, substantial deviations of the results have been demonstrated. Farah et al. (2016) reported ICCs ranging from 0.82 to 0.99, and Plaza-Florido et al. (2020) estimated ICC values between 0.70 and 0.99 [39,40]. Thus, we interpret our ICC results as indicators of high stability.

The reader should keep in mind that MRI and LAB sessions were not in immediate succession but, on average, one week apart. Previous studies have shown that an interval of several days in test–retest studies increases biological variability in HRV indices when compared to immediate retests. Cipryan and Litschmannova (2013) reported a drop in the reproducablity of HR from ICC = 0.94 when measured during the same day to ICC = 0.76 when test–retest sessions were days apart [37]. Biological rhythms or slight differences in the state of health between sessions might be factors influencing HRV stability in our study.

Another methodological influence might arise from the choice of the cardiac signal used for HRV estimation. In the LAB, we acquired ECG and PPG signals simultaneously and estimated HRV based on both recordings. Although HRV measures were well correlated and errors were rather low (MAE: 5.5 ms for SDNN, and 9.1 ms for RMSSD), the Wilcoxon signed-rank test revealed that RMSSD values were systematically higher when calculated using PPG instead ECG signals.

In this analysis, we regarded the ECG as the gold standard for assessing HRV as it captures electrical excitation of the myocardium [36]. Heart beats can be detected with high accuracy since they cause characteristic sharp r-waves in the ECG signal. Ejected blood that travels through the vascular system can be measured via optical sensors at other parts of the body surface such as the fingertip. However, the occurrence of pulse waves is subject to additional influencing factors such as changes in vascular tone or blood pressure [25]. Additionally, the characteristic shape of pulse waves is rather smooth compared to sharp r-waves in an ECG, making an exact temporal detection of pulse onsets difficult.

Although HRV estimated on the basis of PPG signals is widely considered a surrogate for ECG-based HRV [24], some studies have challenged their concordance [26,41]. In a meta-analysis by Schäfer and Vagedes (2013), it was emphasized that HRV, especially short-term HRV, is higher when estimated based on PPG instead of ECG signals [24]. Some authors have suggested pulse rate variability (PRV) as an independent cardiac autonomic marker [25,42]. In a very recent study, Mejía-Mejía and colleagues analyzed 33 HRV indices from different domains derived from ECG and PPG recordings (HRV vs. PRV) [43]. The authors separated a large sample from the MIMIC database into blood pressure categories (hypo-, normo- and hypertensive). Their results suggested that most PRVs from time-domain (including SDNN and RMSSD), frequency-domain and nonlinear indices were higher than their HRV companions. Furthermore, differences between PRV and HRV seemed to be unrelated to blood pressure subgroup. The authors concluded that PRV and HRV were not the same, and especially short-term changes of PRV were overestimated. Those measures, quantifying rapid, high-frequency changes in pulse rate, are particularly sensitive to occasional inaccurate detection of pulse wave onsets. As we also found RMSSD values to be overestimated in terms of PPG analysis, it appears likely that the comparison of HRV between LAB and MRI is affected when analyzing different cardiac signals.

As a psychological factor influencing the agreement of HRV estimates between LAB and MRI, we investigated pre-MRI anxiety. We found a mild but significant correlation of pre-MRI anxiety and the decrease in HRV from LAB to MRI. Although the MRI environment does not always seem to elicit psychological stress, anxiety seems to have an impact on HRV estimates, suggesting that more anxious participants had stronger decreases in HRV during MRI compared with the LAB. However, this effect did not lead to a consistent influence of the MRI condition on HRV indices and might rather be driven by some participants who were extraordinarily agitated and experienced the MRI session as a stressful event. It seems that a familiarization session does not significantly influence the change in HRV outcome between LAB and MRI. However, it should be emphasized here that we did not record ECG signals during MRI. Therefore, a possible effect of anxiety most probably overlaps with the methodological effect of the signal under analysis.

### Limitations

The fact that we cannot separate psychological from methodological effects poses the most significant limitation of this study. We did not acquire ECG signals during MRI sessions to further investigate the influence of the cardiac signal underlying HRV estimation in the scanner. As we did not assess anxiety or agitation prior to LAB sessions, we were not able to quantify their influence on HRV in the LAB. Another limitation is that we used a general inventory to assess pre-MRI anxiety that might be less sensitive than a situation-specific questionnaire in this context.

The reliability of HRV estimates also depends on the processing of cardiac signals and the accuracy of heart beat detection. There is a variety of methods and algorithms available for ECG and PPG analysis. In this study, we presented one approach including the use of a free toolbox for preprocessing and heart beat extraction. This might limit the generalizability of our results. In addition, we kept HRV analysis rather simple, considering the wide range of HRV metrics [44]. Future studies might benefit from more sophisticated and time-resolved analysis of heart rate variations (e.g., [45]).

## 5. Conclusions

We found high reproducibility of HRV during MRI and LAB sessions. However, researchers have to keep in mind that participants’ anxiety might influence the HRV outcome. Furthermore, the choice of a physiological signal to extract HRV is crucial. Researchers should be especially careful when comparing results from pulse recordings with ECG recordings.

## Figures and Tables

**Table 1:** Demographic data of the final sample including 79 healthy volunteers.

Parameter	All Participants	Sample A	Sample B
Age [y]	35 ± 14	38 ± 15	30 ± 10
BMI [kg/m^2^]	24.6 ± 4.0	24.8 ± 4.4	24.4 ± 3.3
Male/Females	36/43	24/31	12/12
Education levels			
8 years of school	3	3	0
10 years of school	12	11	1
12 years of school	33	24	9
University degree	20	16	4
Not disclosed	11	1	10
Anxiety self-ratings			
STAI state	35 ± 8	35 ± 8	39 ± 11
STAI trait	35 ± 9	34 ± 8	43 ± 12
SAM quality	1.8 ± 1.3	1.8 ± 1.4	1.7 ± 0.5
SAM intensity	4.6 ± 2.1	4.7 ± 2.2	4.3 ± 1.4

BMI: body mass index, STAI: State–Trait Anxiety Inventory.

**Table 2:** Comparison of HRV derived from ECG and PPG recorded in the laboratory (N = 55).

HRV Index	LAB_ECG	LAB_PPG	Pearson r	MAE
HR [1/min]	64.6 (57.7–72.3)	64.9 (58.6–72.3)	0.998	0.6
SDNN [ms]	52.5 (38.4–67.6)	52.5 (39.3–65.7)	0.886	5.5
RMSSD [ms]	34.6 (22.7–47.1)	43.2 (28.9–52.7)	0.877	9.1

Descriptive statistics are reported as median value and interquartile range (25–75%). HR: mean heart rate; SDNN: standard deviation of heart beat intervals; RMSSD: root mean square of successive heart beat intervals; LAB_ECG: HRV derived from an electrocardiogram recorded in the laboratory; LAB_PPG: HRV derived from a photoplethysmogram recorded in the laboratory; Pearson r: Pearson correlation coefficient; MAE: mean absolute error.

**Table 3:** HRV indices derived from photoplethysmogram recordings in the laboratory and during MRI compared to electrocardiograms in the laboratory (N = 55).

HRV Index	LAB_ECG	LAB_PPG	MRI_PPG	ICC (95% CI)	CV [%]
HR [1/min]	64.6 (57.7–72.3)	64.9 (58.6–72.3)	67.0 (58.7–76.5)	0.882 (0.808,0.931)	6 ± 5
SDNN [ms]	52.5 (38.4–67.6)	52.5 (39.3–65.7)	52.3 (39.9–61.3)	0.803 (0.681,0.883)	18 ± 15
RMSSD [ms]	34.6 (22.7–47.1)	43.2 (28.9–52.7)	42.9 (31.8–56.0)	0.804 (0.678,0.885)	30 ± 24

Descriptive statistics are reported as median values and interquartile range (25–75%). HR: mean heart rate; SDNN: standard deviation of heart beat intervals; RMSSD: root mean square of successive heart beat intervals; LAB_ECG: HRV derived from an electrocardiogram recorded in the laboratory; LAB_PPG: HRV derived from a photoplethysmogram recorded in the laboratory; ICC: intra-class correlation; CV: coefficient of variation.

**Table 4:** HRV indices derived from repeated recordings of electrocardiograms in the laboratory and photoplethysmogram during MRI (N = 24).

HRV Index	LAB_T0	LAB_T1	MRI_T0	MRI_T1	ICC (95% CI)	CV [%]
HR [1/min]	67.0 (63.3–70.5)	66.2 (62.8–70.6)	68.8 (64.5–76.3)	70.1 (65.7–74.7)	0.796 (0.540,0.927)	5 ± 3
SDNN [ms]	54.5 (41.5–65.9)	57.7 (44.1–65.2)	51.9 (40.1–76.8)	52.4 (36.7–77.5)	0.846 (0.652,0.945)	15 ± 10
RMSSD [ms]	41.1 (33.3–55.8)	46.7 (37.5–56.5)	37.4 (27.5–50.0)	33.8 (26.4–50.8)	0.775 (0.492,0.919)	23 ± 15

Descriptive statistics are reported as median values and interquartile range (25–75%). HR: mean heart rate; SDNN: standard deviation of heart beat intervals; RMSSD: root mean square of successive heart beat intervals; LAB_T0: HRV derived from an electrocardiogram recorded at the first laboratory session; LAB_T1: HRV derived from an electrocardiogram recorded at the second laboratory session; MRI_T0: HRV derived from a photoplethysmogram recorded at the first MRI session; MRI_ T0: HRV derived from a photoplethysmogram recorded at the second MRI session; ICC: intra-class correlation; CV: coefficient of variation.

## Data Availability

No new data were created or analyzed in this study. Data sharing is not applicable to this article.
